# Rapid pathogen identification and phenotypic antimicrobial susceptibility directly from urine specimens

**DOI:** 10.1038/s41598-022-22792-y

**Published:** 2022-11-15

**Authors:** Larry Burg, Gretel Crewe, James DiMeo, Xin Guo, Carmen G. Li, Melissa Mayol, Andrew Tempesta, William Lauzier, Rachelle Markham, Katarzyna Crissy, Colleen Barry, Bruce Walsh, James E. Kirby, Don Straus

**Affiliations:** 1 First Light Diagnostics, 2 Omni Way, Chelmsford, MA 01824 USA; 2 Beth Israel Deaconess Medical Center and Harvard Medical School, Boston, MA 02215 USA

**Keywords:** Laboratory techniques and procedures, Bacterial infection, Microbiology techniques

## Abstract

Implementing effective antimicrobial therapy close to the onset of infection lowers morbidity and mortality and attenuates the spread of antimicrobial resistance. Current antimicrobial susceptibility testing (AST) methods, however, require several days to determine optimal therapies. We present technology and an automated platform that identify (ID) Urinary Tract Infection pathogens in 45 min and provide phenotypic AST results in less than 5 h from urine specimens without colony isolation. The ID and AST tests count cells fluorescently labeled with specific rRNA probes using non-magnified digital imaging. The ID test detected five pathogens at ≤ 7,000 CFU/mL and had a linear range of ~ 4 orders of magnitude. For contrived specimens, AST tests gave 93.1% categorical agreement with 1.3% Very Major Errors (VME), 0.3% Major Errors (ME), and 6.3% minor Errors (mE) compared to the broth microdilution (BMD) reference method. For clinical specimens, the ID test had 98.6% agreement and the AST test had 92.3% categorical agreement with 4.2% mE, 3.4% ME and 4.0% VME compared to BMD. Data presented demonstrates that direct-from-specimen AST tests can accurately determine antimicrobial susceptibility/resistance for each pathogen in a specimen containing two pathogens. The method is robust to urine matrix effects and off-target commensal and contaminating bacteria.

## Introduction

Infectious diseases are the third leading cause of death worldwide^1^. Bacterial infections, which constitute a large fraction of infections, are increasingly difficult to treat using common antibiotics due to the spread of antibiotic resistance. Nearly 3 million antibiotic resistant infections cause about 36,000 deaths and cost about $55B every year in the U.S^2^. Worldwide, 1.27 million deaths were directly attributed to bacterial antimicrobial resistance in 2019^3^. The spread of resistance is exacerbated by the overuse of antibiotics, especially the powerful broad-spectrum drugs. Up to about 50% of antibiotics are used inappropriately: ineffective antibiotics may be prescribed or antibiotics are unnecessarily prescribed to uninfected patients^4^. If current trends continue, an estimated 15-fold increase in deaths due to antimicrobial resistance will occur by 2050^5^.

To quickly cure infections, lower healthcare costs, and attenuate the spread of resistance, patients should ideally be diagnosed and treated close to the onset of symptoms with narrow-spectrum antibiotics targeted specifically to their infecting pathogen. This ideal remains unfulfilled because current culture-based diagnostic methods take 2–3 days to identify causative pathogens and determine the optimal narrow-spectrum therapy. Until these results are available, symptomatic patients are frequently treated empirically with broad-spectrum antibiotics. Empiric therapy with these powerful antibiotics may be unnecessary, suboptimal, or ineffective and may facilitate the spread of antibiotic resistance and lead to complications such as *C. difficile* colitis. In one study, the antibiotic regimen in about 25% of empirically treated UTI patients had to be changed due to resistance to the initial antibiotic^6^.

Conventional pathogen ID and phenotypic AST for syndromic infections (i.e., infections that may be caused by many different pathogens) require multiple steps and several days^7^. Colony isolation, which takes 1–2 days, is required to generate large numbers of pure clonal pathogen cells. Species identification is available in a few hours on automated commercial instruments (e.g., Bruker MALDI Biotyper) or in 1–2 days for biochemical tests (e.g., bioMerieux API tests). Following pathogen ID, AST results are usually available in about 4–10 h on automated commercial systems (e.g., bioMerieux VITEK 2) or in 18 h for the gold-standard broth microdilution (BMD) reference method or disk diffusion. For specimens containing a mixture of species, AST systems using non-specific detection methods (e.g., turbidity) cannot determine which species are growing and which are not and therefore require colony isolation so input cells are of a single, pure strain.

Non-phenotypic molecular technologies are especially effective for identifying the pathogen species and detecting resistance genes directly from specimens. Species identification alone does not, in general, determine which antibiotics are effective since various strains of a species can have different antibiotic susceptibilities. Antimicrobial resistance can be inferred, but is not certain, by detecting resistance genes using molecular methods—these are the antibiotics the clinician *should not* use. This contrasts with growth-based phenotypic methods which indicate to which antibiotics the pathogen is susceptible—that is, which antibiotics the clinician *can* use. Next generation sequencing methods are also being developed for AST, but these methods may be too inaccurate, slow, complex, and expensive for routine clinical testing^8^.

Several emerging platforms can deliver AST results faster than traditional culture methods. These new methods speed up the detection of pathogen growth by using technologies based on microscopy, biosensors, fluorescence-based measurement of surface area, and nucleic acid quantification^9–12^. Most of these methods, while providing significantly faster results than current phenotypic AST methods, still require the long colony isolation step to provide high numbers of pure cells. Several new methods have the potential to test specimens directly without colony isolation because, like the method presented here, they measure growth of specific pathogens in the clinical specimens^13,14^.

This work addresses rapid ID and AST methods for urinary tract infections (UTI), one of the most common infections and responsible for about 9 million illnesses and about $3.5B in healthcare costs each year in the US^15,16^. Most UTIs are classified as acute uncomplicated cystitis—these are common in healthy young women without urinary tract-related comorbidities. Complicated UTIs are those that occur in men or patients suffering from recurrent UTIs, with in-dwelling catheters, or functional or anatomical abnormalities. Catheter-associated UTI (CAUTI) is the most common hospital acquired infection and is the root cause of about 25% of blood infections^17,18^. In the absence of timely UTI diagnostic results, uninfected patients are often treated unnecessarily with empiric antibiotic therapy, leading to the spread of resistance.

MultiPath® technologies—single-molecule counting, non-magnified digital imaging, and use of a dye-cushion to avoid wash steps—were previously shown to detect *C. difficile* toxin B with high sensitivity and specificity using magnetic and fluorescent particles conjugated with specific antibodies^19^. The work presented herein extends those MultiPath technologies to include an automated analyzer, single-use cartridges, non-specific magnetic tagging of bacteria, and 1-step fluorescent labeling of bacterial cells by fluorescent in situ hybridization (FISH) to specific rRNA sequences. This system enables the detection, identification, and antimicrobial susceptibility determination of the most common pathogens causing UTIs, directly from the primary urine specimen in less than 6 h without the need for lengthy colony purification.

## MultiPath automated platform and rapid bacterial ID and AST methods

### MultiPath automated platform

**Figure 1:**
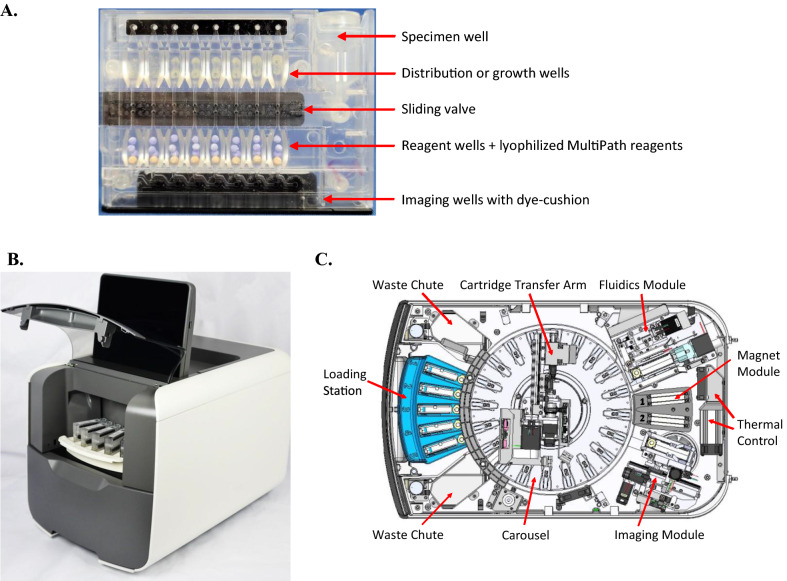
MultiPath cartridge and analyzer. (**A**) Picture of a MultiPath UTI cartridge showing the key features. (**B**) Exterior view of the MultiPath benchtop analyzer showing the cartridge loading bay and user interface touchscreen. Dimensions are 15″W × 25″D × 23″H. (**C**) Top view schematic of the interior of the MultiPath analyzer showing the major modules.

The MultiPath technology and *C. difficile* toxin test described previously^19^ have been configured into single-use test cartridges which are automatically processed on the MultiPath analyzer (Fig. 1). Both the MultiPath analyzer and *C. difficile* test have been cleared by the FDA. MultiPath rapid bacterial ID and phenotypic, growth-based AST tests for UTIs have now been designed for this automated platform.

Test cartridges (Fig. 1A) have 8 or 16 independent reaction channels containing dried media plus antibiotics in the growth wells (AST tests only), lyophilized FISH and magnetic particle beads in the reagent wells and dried dye-cushion in the imaging wells. ID and AST tests are performed using the processed urine specimen as the only fluid to rehydrate reagents; washing steps are avoided by the dye-cushion which reduces background fluorescence^19^.

Up to 20 cartridges can be processed on the MultiPath analyzer (Fig. 1B,C) with continuous random-access capability. Specimen is moved through the cartridge in the fluidics module using positive or negative pressure and positioning the valve as necessary. Incubation occurs at 35 °C in the carousel which also has a cartridge transfer arm to shuttle cartridges to and from the various modules. Fluorescently labeled and magnetically tagged cells are pulled to the bottom of imaging wells in the magnetics module where they are subsequently detected and quantified in the imaging module. MultiPath software provides the user interface, controls all analyzer functions using test-type cartridge barcodes for appropriate parameters, analyzes images, and reports final calculated results.

### MultiPath technology for rapid bacterial ID and AST from primary urine specimens

Bacterial cells are first isolated directly from 2 mL urine specimens, without colony isolation, using a 3-min size exclusion chromatography (SEC) method carried out in a column separate from the cartridge. Isolated cells are recovered in optimal conditions for subsequent steps to normalize diverse compositions of urine (e.g., NaCl and pH). Initial studies showed SEC also removed > 99% of boric acid preservative and antibiotics (e.g., ciprofloxacin, cefepime, trimethoprim/sulfamethoxazole, meropenem), which otherwise would interfere with bacterial growth (data not shown).

SEC-processed urine is then tested in MultiPath ID or AST assays using either cartridges and the MultiPath Analyzer (automated format) or microwell plates followed by image acquisition in a custom imager (manual format). Both formats use the same fluorescence optics, software, and parameter settings to acquire images. Analysis of digital images and algorithm calculations of results are automatically performed on the analyzer for cartridge tests or separately with identical stand-alone software for images acquired from microwell plates tests.

**Figure 2:**
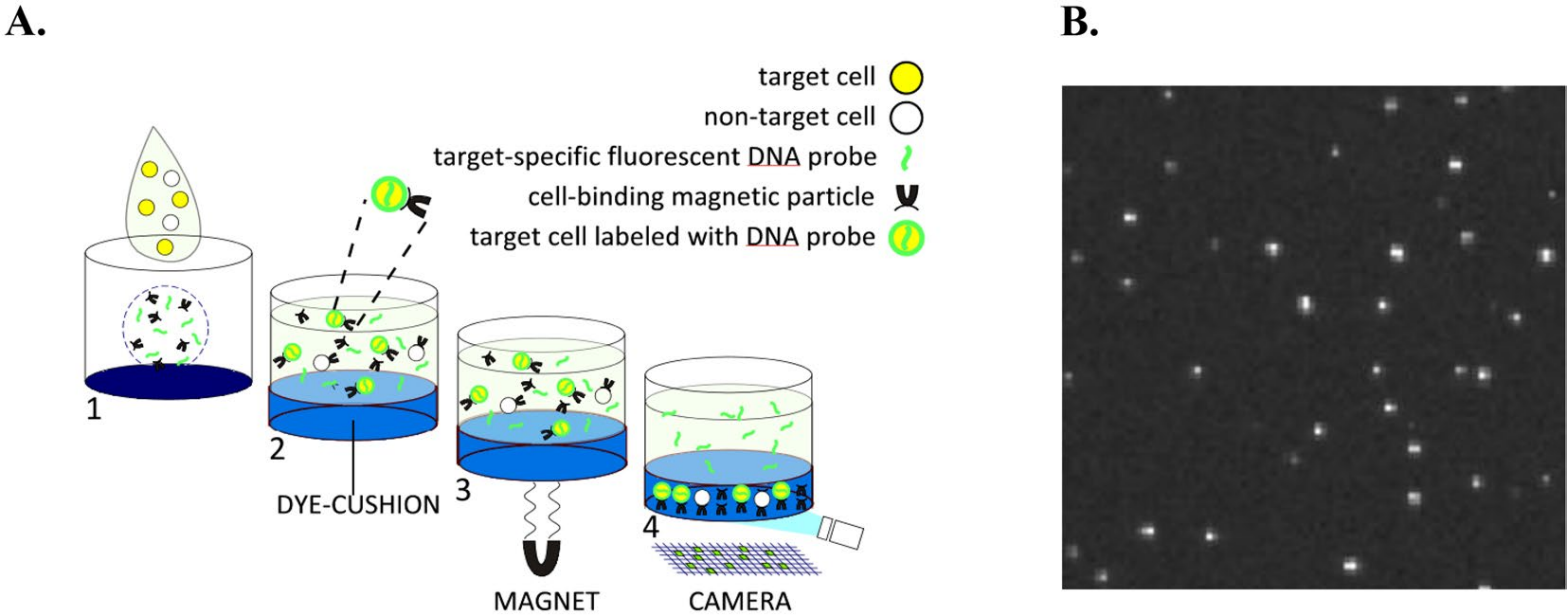
Multipath FISH assay for detecting specific target pathogens. (**A1**) Specimen is added to lyophilized FISH reagents, lyophilized cell-binding magnetic particles plus fluorescent magnetic focusing particles, and a dried, opaque dye-cushion. (**A2**) Following contact with the specimen, reaction components are rehydrated to initiate the 1-step reaction: cell permeabilization, probe hybridization to specific target rRNA sequences and magnetic particle binding to cells. (**A3**) Following a 30-min reaction, magnetically tagged cells are pulled through the dye-cushion to the imaging well’s clear bottom. (**A4**) A non-magnified digital image of the labeled cells is taken. (**B**) Zoomed-in black and white image showing individual labeled *K. pneumoniae* cells.

**Figure 3:**
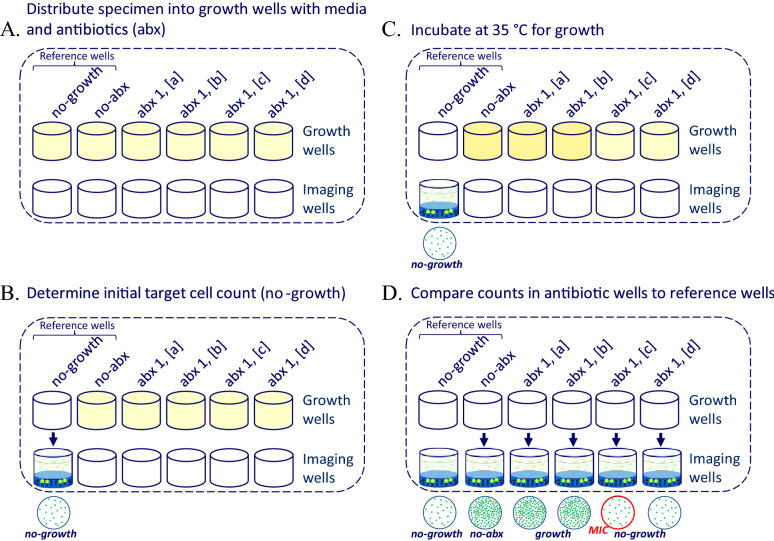
Principle of MultiPath AST assays. (**A**) A processed urine specimen is distributed to wells containing dried growth media and antibiotics, except for the no-growth and no-abx reference wells which do not have antibiotics. One antibiotic is shown (abx 1) at 4 increasing concentrations from [a] to [d]. (**B**) The no-growth reference sample is moved to its imaging well to quantify input cells, as shown in Fig. 2, prior to incubation. (**C**) Bacterial growth occurs at 35 °C for a predetermined amount of time, typically 4 h for rapidly dividing bacteria. (**D**) Post-incubation samples are moved to imaging wells to quantify cells. Cell counts are compared to the no-growth or no-abx reference wells to determine whether growth occurred. The MIC in this case is shown in red at abx1 [c]. By measuring growth in multiple antibiotic concentrations near CLSI breakpoints or determining MICs, categorical susceptibility (SIR) can be easily determined.

MultiPath principles for bacterial ID and AST assays are shown schematically in Figs. 2 and 3 and described in detail in the Materials and Methods section. The MultiPath bacteria detection and quantitation method, used for both ID and AST assays, is based on a 30-min, 1-step, isothermal reaction where cells are permeabilized, fluorescently labeled with Alexa647 probes (Ex: 630 nm, Em: 680 nm) to species-specific 16S or 23S rRNA sequences and bound to paramagnetic particles using electrostatic interactions (Fig. 2A1,A2). Following incubation at 35 °C, the structurally intact cells are magnetically captured onto the clear bottom of the imaging well. Wash steps are not required to remove unhybridized probe and auto-fluorescent molecules in residual specimen because an opaque, dense, dye-cushion prevents these from reaching the bottom surface of the reaction well. Finally, a non-magnified digital image is taken of the entire well in about 1 s, in which only the specifically targeted cells are fluorescently labeled and thus detected (Fig. 2A3,A4). Fluorescent magnetic focus particles (Ex: 470 nm, Em: 515 nm; not shown in Fig. 2) are used to establish the focal plane. Figure 2B shows a zoomed-in portion of a resulting MultiPath image, where individually labeled *K. pneumoniae* cells can be seen and quantified. A single 16-channel MultiPath ID cartridge can be configured to separately detect 14 specific bacterial pathogens plus internal positive and negative controls in 45 min.

If the MultiPath ID test detects a target pathogen at a level above the clinical threshold of 1 x 10^4 ^CFU/mL, its antibiotic susceptibility is determined in a separate pathogen-specific MultiPath AST cartridge using the same SEC-processed specimen. The MultiPath AST principle is illustrated in Fig. 3 and is essentially the MultiPath ID test just described preceded by a bacterial growth step in the presence or absence of antibiotics. A no-growth reference is included to quantify the input cell inoculum. Samples are then incubated with media at 35 °C without antibiotic (no-abx reference) or with different antibiotics at different concentrations. Post-incubation cell counts are obtained, using the same FISH-based ID methods, and cell counts in reactions with antibiotics are compared to the no-growth or no-abx references. Antibiotic concentrations that prevent growth will have cell counts much lower than the no-abx reference and more like the no-growth reference. In contrast, antibiotic concentrations that do not prevent growth will have cell counts similar to the no-abx reference and significantly greater than the no-growth reference. By comparing growth outcomes to antibiotic concentrations, MICs can be determined and compared to CLSI or FDA breakpoints to establish categorical susceptibility (i.e., SIR). Although the MultiPath AST method can determine MIC results as illustrated in the figure and as reported in some experiments in microwell plates described in sections below, the automated, cartridge-based platform is primarily designed to report only categorical results using a few antibiotic concentrations near the breakpoints.

## Results

### Correlation of MultiPath cell detection and input pathogen cells

**Figure 4:**
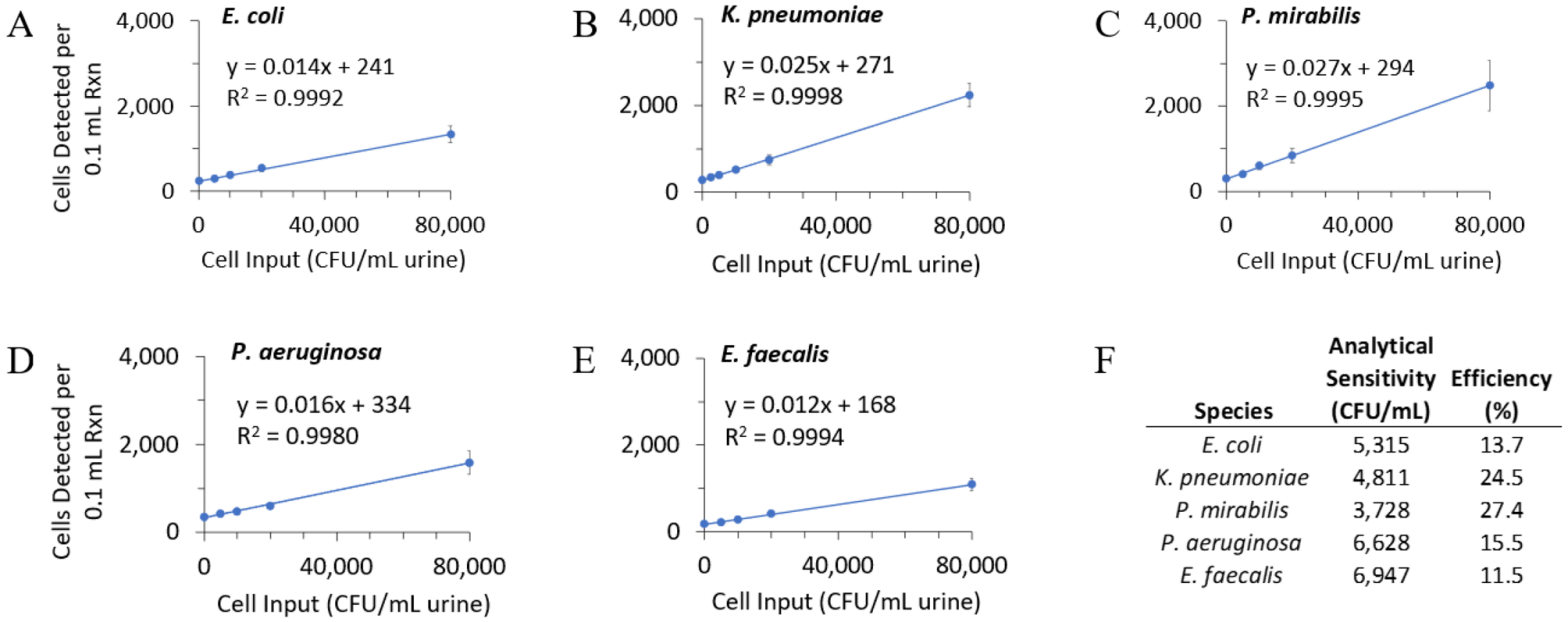
Correlation between MultiPath signal and input cells. Bacterial cells were added to pooled urine from 0 to 80,000 CFU/mL, processed by rapid SEC, added to test cartridges, and automatically processed on the MultiPath analyzer. (**A**–**E**) Ordinary least squares regression models and coefficients of determination for 5 MultiPath bacterial assays as indicated. Input cell concentrations were estimated from OD_600_ measurements of broth cultures prior to dilution in urine. Plotted data points are means of 6 or 7 replicates per cartridge and 3–5 processed cartridges per species. Error bars are plus/minus one SD and in many cases are too small to be seen. (**F**) Analytical sensitivity and assay efficiency for each of the 5 assays.

Bacterial pathogens in urine are typically considered clinically relevant for patients suspected of UTI if they are present at concentrations above 1 × 10^4^ CFU/mL. The correlation between input pathogen concentration in CFU/mL and MultiPath cell detection was determined by spiking urine with 5 of the most common uropathogens from 0 to 8 × 10^4^ CFU/mL, processing the samples by rapid SEC, and testing them in cartridges on the MultiPath analyzer (Fig. 4). Each concentration of each species was tested in 3–5 cartridges (each with a separate SEC cell isolation step) containing 6 or 7 technical replicate reactions per cartridge. The bacteria strains tested were *E. coli* ATCC 25922, *K. pneumoniae* ATCC 13883, *P. mirabilis* ATCC 7002, *P. aeruginosa* ATCC 27853 and *E. faecalis* CDC 580.

MultiPath results for all 5 pathogens linearly correlated with input cell concentration up to 8 × 10^4^ CFU/mL with coefficients of determination (i.e., R^2^) ≥ 0.998. The data also allowed for an estimation of assay analytical sensitivity, which was less than 7,000 CFU/mL in all cases (see Materials and Methods for calculations). Assay precision was also estimated, with CVs at 5,000 CFU/mL input of 4.3%, 8.3%, 10.4%, 8.6% and 11.9% for *E. coli* (n = 21), *K. pneumoniae* (n = 16), *P. mirabilis* (n = 24), *P. aeruginosa* (n = 24) and *E. faecalis* (n = 24), respectively.

Finally, the slopes of the linear regression models are related to the efficiency of the MultiPath ID reactions where the number of cells detected by MultiPath per 0.1 mL of post-processed urine are correlated to input cells per mL of pre-processed urine. The efficiencies shown in Fig. 4F, corrected by a factor of 10 to account for the different units of volume for input cells (CFU per 1 mL) and MultiPath results (cells detected in 0.1 mL reaction volumes), are similar for the 5 target pathogens. Assay efficiencies below 30% may be attributable to several factors. Cell loss during urine processing by SEC (comparing colony counts pre- and post-processing) accounts for about 10% to 20% of the loss of assay efficiency. Lysis of roughly 30% of cells may occur, estimated by microscopic evaluation, due to over-permeabilization. The remaining about 20% to 30% of efficiency loss is accounted for by incomplete detection of the remaining structurally intact cells. This may be caused by a combination of incomplete probe hybridization causing a distribution of fluorescent cell intensities, incomplete magnetic cell capture and technical imaging considerations (e.g., energy loss by optical bandwidth filters, quenching, quantum efficiency of fluorophores, exposure time, image normalization, fluorescent background, properties of CMOS sensor, and threshold settings). Preliminary work has shown efficiency gains are possible through optimizing assay reagent formulations, decreasing background, using a higher resolution CMOS detector, and adding additional fluorescent probes.

### Analytical measuring interval

**Figure 5:**
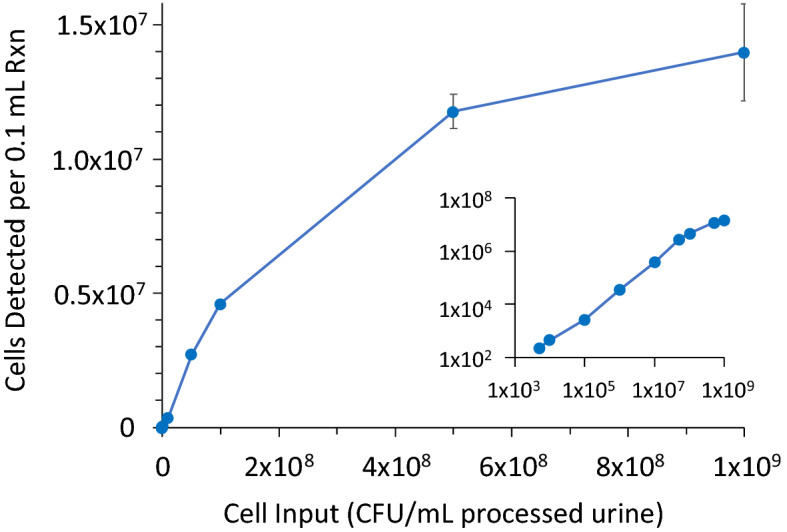
Analytical measuring interval of MultiPath *E. coli* assay. *E. coli* ATCC 25922 cells were grown to mid-log phase, concentrated, and diluted in SEC-processed urine at concentrations ranging from 5 × 10^3^ to 1 × 10^9^ CFU/mL. Detected cells were estimated from total fluorescence. Plotted data points are means of triplicate reactions. Error bars represent +/− 1 SD. The inset shows a log–log plot so all data points can be seen.

Whereas the correlation studies centered on input cell concentrations at the limit of sensitivity and near the clinical cutoff of 1 × 10^4^ CFU/mL, determination of MultiPath ID method’s analytical measuring interval focused on the range of cell inputs that can be measured. The analytical measuring interval of the MultiPath ID technology was estimated using the *E. coli* assay in the microwell format using lyophilized reagents. *E. coli* strain ATCC 25922 was grown to mid-log phase in MHB II media, rapidly chilled to arrest metabolism, centrifuged to pellet cells and resuspended in MHB II media to concentrate cells 125-fold. Concentrated cells were then diluted in SEC-processed urine to concentrations ranging from 5 × 10^3^ to 1 × 10^9^ CFU/mL and tested in triplicate (Fig. 5).

No hook effect (i.e., loss of signal) was seen up to 1 × 10^9^ CFU/mL, the approximate maximum cell concentration in a typical broth culture. The inset shows a dynamic range of about 5 orders of magnitude (about 5 × 10^3^ to 5 × 10^8^ CFU/mL) and a linear range of about 4 orders of magnitude (about 5 × 10^3^ to 5 × 10^7^ CFU/mL; R^2^ = 0.996 for linear regression). Loss of linear proportionality between cell input and MultiPath signal, beginning above about 5 × 10^7^ CFU/mL is most likely caused by changing reaction kinetics as key reactants (e.g., fluorescent probes, magnetic particles) start to become limiting. The large dynamic range is made possible by algorithms that estimate detected cells from total fluorescence—using the average fluorescence per cell—when image density increases to the point where individual fluorescent objects merge and can no longer be distinguished. The MultiPath assays are therefore sufficiently configured to cover the clinically relevant range of pathogen concentrations in urine and the high bacterial concentrations following growth in AST testing.

### Analytical specificity (inclusivity and exclusivity)

MultiPath ID and AST assays depend greatly on high specificity since UTIs may be caused by many different species and strains of those species. Furthermore, the direct-from-specimen approach means specimens may contain multiple bacterial species, whether pathogenic or not. The specificity of MultiPath ID assays was therefore challenged in a series of studies that focused on: (1) the ability to detect multiple strains of the target pathogens; (2) the absence of cross-reactivity with non-target pathogens; and (3) the ability to detect the target pathogen in the presence of non-target bacteria.

Multiple strains of three common uropathogens were tested in MultiPath assays to ensure that clinically relevant and diverse strains can be detected (Table 1). Various strains (isolates from different patient specimens and culture collections) of *E. coli* (n = 97), *K. pneumoniae* (n = 49), and *E. faecalis* (n = 36) were spiked into PBS at a concentration of about 1 × 10^5^ CFU/mL and tested in MultiPath assays (n = 1). Every strain tested was detected except for one *K. pneumoniae* strain.

**Table 1:** Inclusivity of MultiPath assays.

Species	# Strains tested	# Strains detected
*E. coli*	97	97
*K. pneumoniae*	49	48
*E. faecalis*	36	36

Uropathogen-specific MultiPath assays were carried out in PBS spiked with about 1 × 10^5^ CFU/mL of various strains. Strains were obtained from commercial sources, provided by Assurance Scientific Laboratories or isolated from UTI patients’ urine collected under an IRB approved protocol.

The analytical specificities of the MultiPath ID assays for the 5 common uropathogens shown in Fig. 4 were determined by testing 15 off-target bacterial species (n = 3) spiked into urine at 5 × 10^6^ to 1 × 10^7^ CFU/mL and processed by SEC (Table 2). No-cell controls were also tested in triplicate for the 5 assays. For off-target bacteria, MultiPath results were similar to no-cell controls for all test combinations, except for the *K. pneumoniae* test, which showed minor cross-reactivity with *S. marcescens* cells. This minor cross-reactivity was subsequently eliminated by further optimizing the NaCl concentration in the *K. pneumoniae* FISH reagent (data not shown). Collectively, these results show MultiPath assays are designed and formulated to detect only the intended target bacteria.

**Table 2:** Specificity of MultiPath ID assays.

Bacteria	Strain	MultiPath ID Tests				
		*E. coli*	*K. pneumoniae*	*P. mirabilis*	*P.aeruginosa*	*E. faecalis*
*E. coli*	ATCC 25922	++++	−	−	−	−
*K. pneumoniae*	ATCC 13883	−	++++	−	−	−
*P. mirabilis*	ATCC 7002	−	−	++++	−	−
*P. aeruginosa*	ATC 27853	−	−	−	++++	−
*E. faecalis*	ATCC 29212	−	−	−	−	++++
*S. saprophyticus*	ATCC 43867	−	−	−	−	−
*A. baumannii*	ATCC 19606	−	−	−	−	−
*S. agalactiae*	ATCC 13813	−	−	−	−	−
*E. cloacae*	ATCC 35030	−	−	−	−	−
*S. marcescens*	ATCC 8100	−	+ /−	−	−	−
*M. morganii*	ATCC 25830	−	−	−	−	−
*C. koseri*	CDC 24	−	−	−	−	−
*C. freundii*	ATCC 8090	−	−	−	−	−
*K. aerogenes*	ATCC 13048	−	−	−	−	−
*K. oxytoca*	ATCC 13182	−	−	−	−	−
*S. aureus*	ATCC 29213	−	−	−	−	−

16 bacterial species were individually added to pooled urine at 5 × 10^6^ to 1 × 10^7^ CFU/mL, processed by SEC and tested in triplicate with the 5 MultiPath tests in the manual plate format. No-cell samples were tested in triplicate as controls. Specific signals, indicated by “++++” averaged about 136,000 above the no-cell controls. In contrast, non-specific signals, indicated by “−”, were similar to no-cell values (average signal of -7). The single cross-reactivity between the *K. pneumoniae* test and *S. marcescens* cells, shown with a “ + /−”, had a signal of 1,813 relative to the no-cell control.

**Figure 6:**
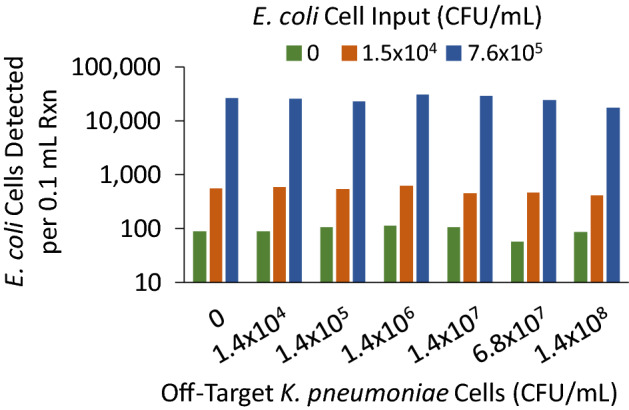
Specific cell detection is not affected by excess of off-target bacteria. *E. coli* strain ATCC 25922 was tested by the MultiPath *E. coli* test in the presence of *K. pneumoniae* ATCC 13883 cells at the concentrations shown. Matrix was SEC-processed urine and the test carried out in microwell plates with lyophilized reagents. Bars represent the average of duplicate reactions. Detected cells for 7.6 × 10^5^ input *E. coli* was estimated from total fluorescence.

The specific detection of a targeted pathogen in the presence of an excess of off-target bacteria was also demonstrated (Fig. 6), an important finding since magnetic cell capture is a non-specific step. In this experiment, the MultiPath *E. coli* ID assay was tested with samples prepared in processed urine with 0, 1.5 × 10^4^ or 7.6 × 10^5^ CFU/mL *E. coli* ATCC 25922 in the presence of *K. pneumoniae* ATCC 13883 cells ranging from 0 to 1.4 × 10^8^ CFU/mL. Neither the no-cell background nor the specific detection of *E. coli* cells was substantially affected by off-target *K. pneumoniae* cells, even when present in about a 10,000-fold excess.

### MultiPath AST robustness to urine matrix

As a preliminary test of the MultiPath AST method’s robustness to matrix effects, 10 diverse urine samples were processed by SEC and spiked with 5 × 10^5^ CFU/mL of *E. coli* ARLG 1042 (susceptible to nitrofurantoin) or *E. coli* ARLG 1175 (resistant to nitrofurantoin). MICs to nitrofurantoin (range 4–512 µg/mL) were determined by MultiPath AST tests (n = 2) using lyophilized reagents and the manual microwell format (Table 3). One fresh urine was obtained from a consenting donor who was taking 100 mg nitrofurantoin twice daily for a UTI infection. The susceptible strain gave MICs of 32 µg/mL for all 10 urine specimens and the BMD reference. The resistant strain gave MICs of 128 µg/mL for all 10 urine matrices and 256 µg/mL for the BMD reference (within essential agreement). Assuming high levels of nitrofurantoin were concentrated in the UTI donor’s urine as usual, it had no effect on the MIC accuracy (nitrofurantoin is effectively removed by the SEC urine processing step).

**Table 3:** MultiPath AST robustness to urine matrix.

Urine matrix	E. colli ARLG1042	E. coii ARLG1175
	(NIT susceptible)	(NIT resistant)
None (BMD reference method)	32 µg/mL	256 µg/mL
Human pool, Lot 34,263	32	128
1st trimester pregnancy donor	32	128
2nd trimester pregnancy donor	32	128
3rd trimester pregnancy donor	32	128
Type 1 diabetic donor	32	128
Healthy donor 1	32	128
Healthy donor 2	32	128
Healthy donor 3	32	128
Healthy donor 4	32	128
Fresh urine from UTI patient taking nitrofurantoin (100 mg BID)	32	128

Urine from a variety of donors was processed, spiked with 5 × 10^5^ CFU/mL of a susceptible or resistant *E. coli* strain, and tested with the MultiPath *E. coli* AST assay for susceptibility to nitrofurantoin. There was no difference between urine matrices and all MICs were within essential agreement to the BMD reference method. CLSI breakpoints for *E. coli* with nitrofurantoin are S ≤ 32, I = 64, R ≥ 128 µg/mL.

### MultiPath AST accuracy in the presence of commensals

**Figure 7:**
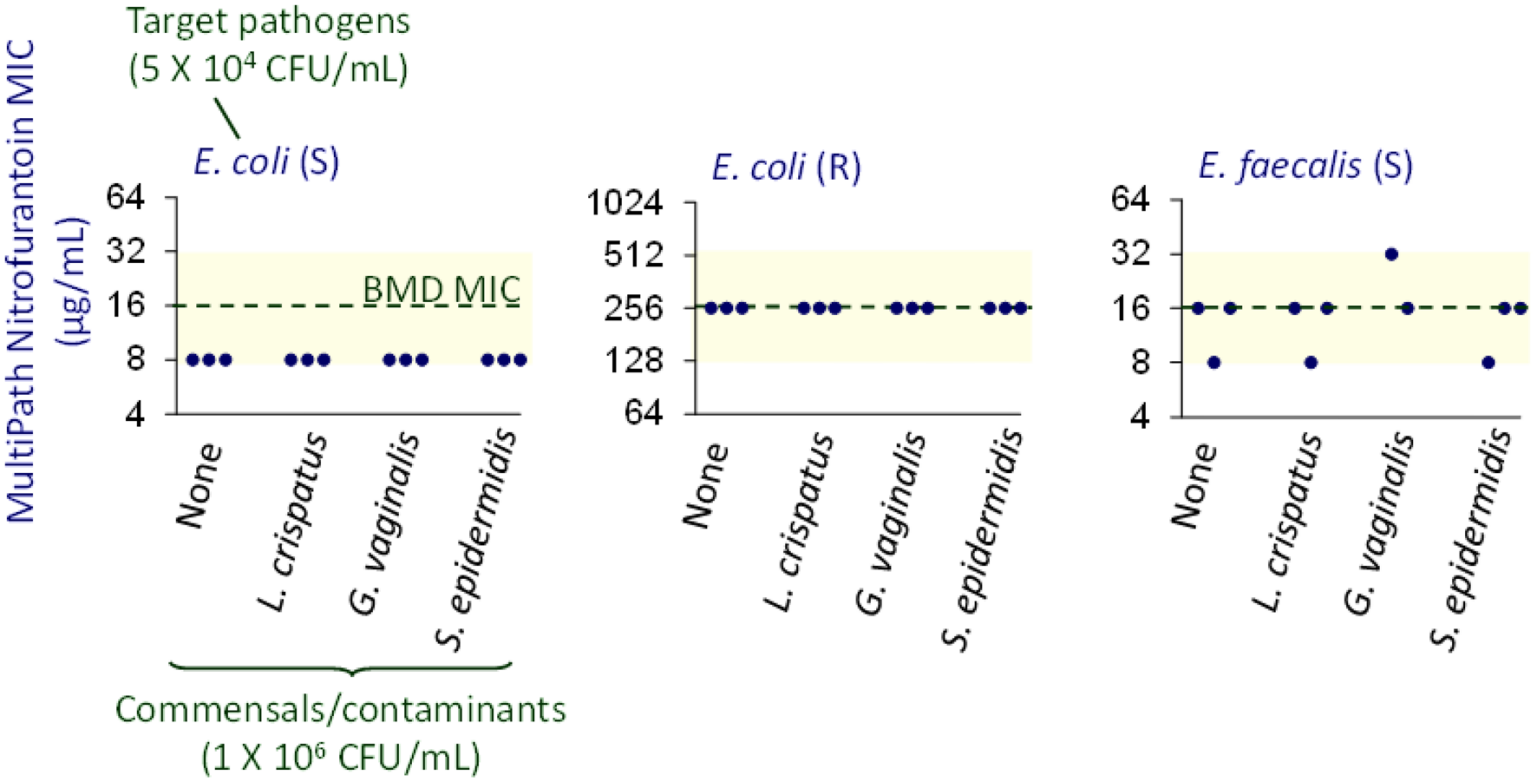
MultiPath AST accuracy in the presence of commensal and contaminating bacteria. Nitrofurantoin susceptibility results (MIC) for 3 target uropathogens either alone or with a urine commensal or contaminating bacteria as shown. Target strains were *E. coli* ARLG 1050 (S), *E. coli* BI 703 (R) and *E. faecalis* CDC 580 (S). Samples were contrived in SEC-processed urine. The dashed line denotes the MIC result for the target pathogen found using the BMD reference method. The yellow shaded area shows the limits of essential agreement with the BMD method. CLSI breakpoints for these pathogens with nitrofurantoin are S ≤ 32, I = 64, and R ≥ 128 µg/mL.

The potential of MultiPath AST assays to determine accurate antimicrobial susceptibility for pathogens in urine specimens that also harbored one of several common commensals or contaminating bacteria was evaluated (Fig. 7). Two *E. coli* strains, one susceptible and one resistant to nitrofurantoin, and one resistant *E. faecalis* strain, were spiked into SEC-processed urine either alone or with one of three bacteria commonly found in urine (*Lactobacillus crispatus*, *Gardnerella vaginalis*, and *Staphylococcus epidermidis*)^20^. MultiPath AST results (MICs) of the targeted bacteria were compared with and without the off-target bacteria and to MICs obtained by the BMD reference method. For this study, 5 × 10^4^ CFU/mL of the target pathogens were tested alone or in the presence of 1 × 10^6^ CFU/mL of the off-target species (n = 3). The experiment was conducted in microwell plates in the manual format. As shown in the figure, the MultiPath MICs were not impacted by the presence of the commensal or contaminating bacteria. The MultiPath SIR and MIC results also agreed well with the gold-standard BMD reference method.

### *MultiPath AST accuracy for two pathogens* in the same specimen

**Figure 8:**
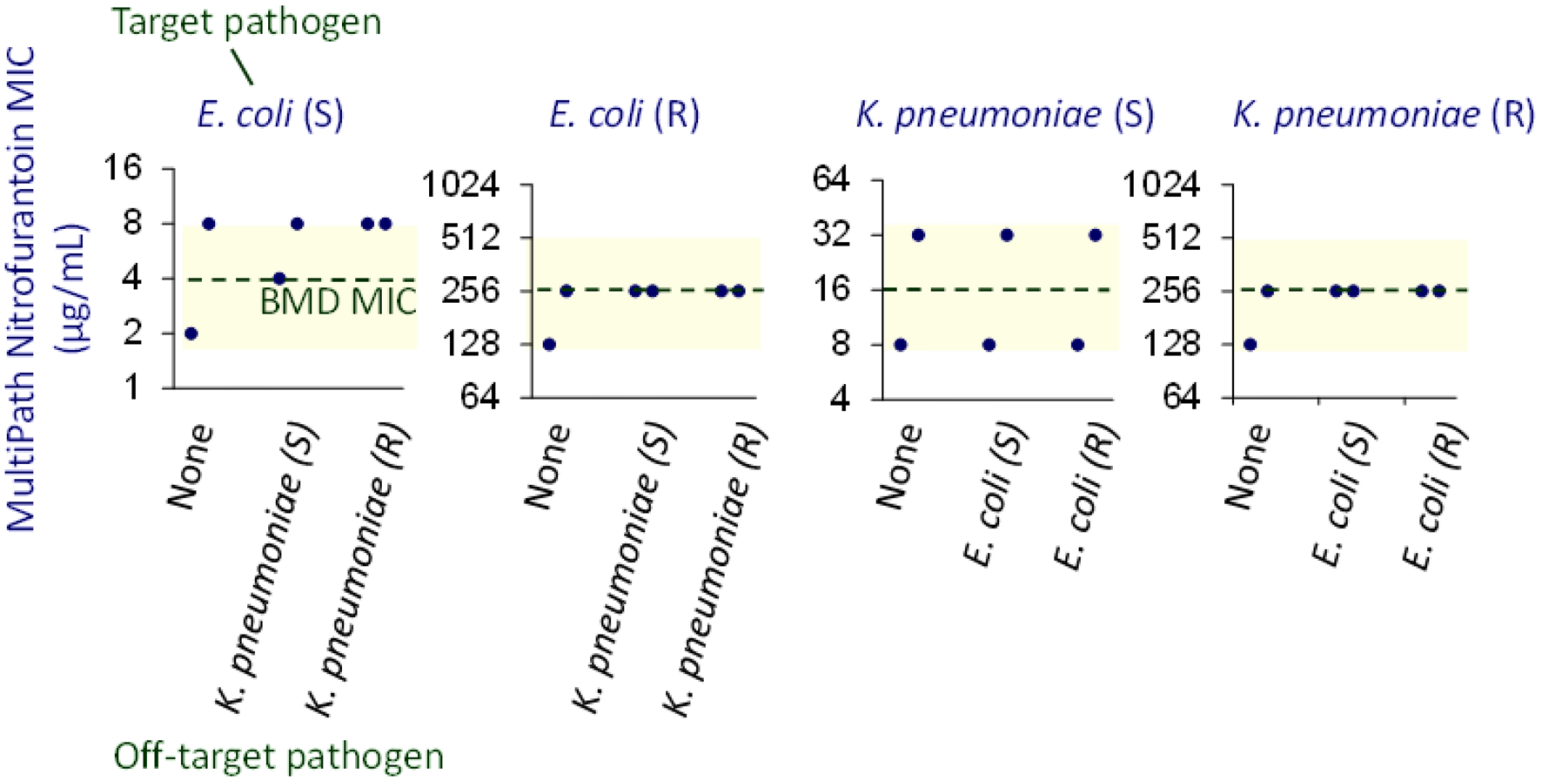
MultiPath AST accuracy for multiple pathogens in a sample. MultiPath nitrofurantoin susceptibility results (MIC) are shown for each pathogen in a sample containing two pathogen species compared to the result for that pathogen alone (n = 2). The strains were *E. coli* ARLG 1021 (S), *E. coli* BI 703 (R), *K. pneumoniae* CDC 142 (S), and *K. pneumoniae* CDC 555 (R). The dashed line denotes the MIC result for the target pathogen found using the BMD reference method. The yellow shaded area shows the limits of essential agreement with the BMD method. CLSI breakpoints for these pathogens with nitrofurantoin are S ≤ 32, I = 64, R ≥ 128 µg/mL.

The potential for MultiPath AST tests to accurately determine antimicrobial susceptibility for two uropathogens in a single specimen (i.e., a polymicrobial infection) was investigated (Fig. 8). Four samples were contrived in pooled urine at 5 × 10^4^ CFU/mL, each containing a susceptible or resistant *E. coli* strain paired with a susceptible or resistant *K. pneumoniae* strain; the antibiotic was nitrofurantoin. These 4 pairwise samples were tested in duplicate with both the MultiPath *E. coli* and *K. pneumoniae* AST assays. Controls were MultiPath AST tests with each of the 4 bacteria alone and BMD reference results.

The results show that the MultiPath *E. coli* and *K. pneumoniae* AST assays accurately determine MIC and categorical susceptibilities of specimens containing both *E. coli* and *K. pneumoniae* cells regardless of the pairwise combinations of susceptible and resistant phenotypes. The AST results of the 4 polymicrobial samples agreed with the strains alone and with the BMD reference results.

### MultiPath AST accuracy using contrived specimens

To test the accuracy of rapid MultiPath AST assays, panels of *E. coli*, *K. pneumoniae*, and *E. faecalis* strains were tested with several different classes of antibiotics and compared to the BMD reference method (Table 4). Samples were tested using the manual microwell format at 1 × 10^5^ or 1 × 10^6^ CFU/mL in PBS with 4 and 6 h of growth at 35 °C (a subset of strains were also grown for 2 or 3 h, but this proved an insufficient amount of growth; data not shown). A custom software tool allowed selection of the best thresholds to determine whether a reaction in the presence of antibiotic was deemed ‘growth’ or ‘no-growth’ relative to the number of cells detected without antibiotic. These thresholds varied depending on the bacteria-antibiotic combination and were typically set in the range of 8–30% relative growth. Maximal concordance was seen at both 4 and 6 h of growth.

**Table 4:** MultiPath AST accuracy compared to the BMD reference method.

Experimental Condition			BMD Reference			MultiPath vs BMD Reference			
Bacteria	# Strains	Drug	S	I	R	CA	mE	ME	VME
*E. coli*	97	CPD	66	1	30	92	4	0	1
	99	NIT	88	2	9	93	5	0	1
	50	SXT	34	n/a	16	50	n/a	0	0
*K. pneumoniae*	49	CPD	17	0	32	48	0	1	0
	48	NIT	7	9	32	38	10	0	0
	49	SXT	21	n/a	28	49	n/a	0	0
gram-negative summary	Results	392	233	12	147	370	19	1	2
	Results (%)	100	59.4	3.1	37.5	94.4	4.8	0.4*	1.4^†^
*E. faecalis*	36	LZD	28	6	2	27	9	0	0
	36	NIT	35	1	0	35	1	0	0
gram-positive summary	Results	72	63	7	2	62	10	0	0
	Results (%)	100	87.5	9.7	2.8	86.1	13.9	0.0*	0.0^†^

MultiPath samples were contrived in PBS with various strains as indicated at 1 × 10^5^ CFU/mL (*E. coli, K. pneumoniae*) or 1 × 10^6^ CFU/mL (*E. faecalis*). MultiPath results were generated in microwell AST assays using 4 h of growth and analyzed using relative growth in the presence and absence of antibiotic. S, I and R are susceptible, intermediate, and resistant, respectively. CA, mE, ME and VME are abbreviations for categorical agreement, minor error, major error (false resistance) and very major error (false susceptibility), respectively. *ME percentage is calculated relative to BMD susceptible results. †VME percentage is calculated relative to BMD resistant results. n/a is not applicable; CPD = cefpodoxime; NIT = nitrofurantoin; SXT = trimethoprim-sulfamethoxazole; LZD = linezolid.

A comparison of the categorical AST results for the MultiPath AST and BMD AST methods is shown in Table 4. For the two gram-negative species with 3 antibiotics, there was 94.4% categorical agreement, 4.8% mE, 0.4% ME and 1.4% VME. For the gram-positive *E. faecalis* with 2 antibiotics, there was 86.1% categorical agreement with 13.9% mE, no ME and no VME. The n-size was small for *E. faecalis*; in particular there were just two resistant results. Combining all species and antibiotics, MultiPath AST tests had 93.1% categorical agreement, 6.3% mE, 0.3% ME and 1.3% VME compared to the gold-standard BMD method. These results show the potential of the MultiPath AST technology for delivering accurate and rapid AST results for both gram-negative and gram-positive species and a variety of antibiotic classes.

### Clinical performance studies

#### MultiPath ID accuracy in clinical specimens

**Figure 9:**
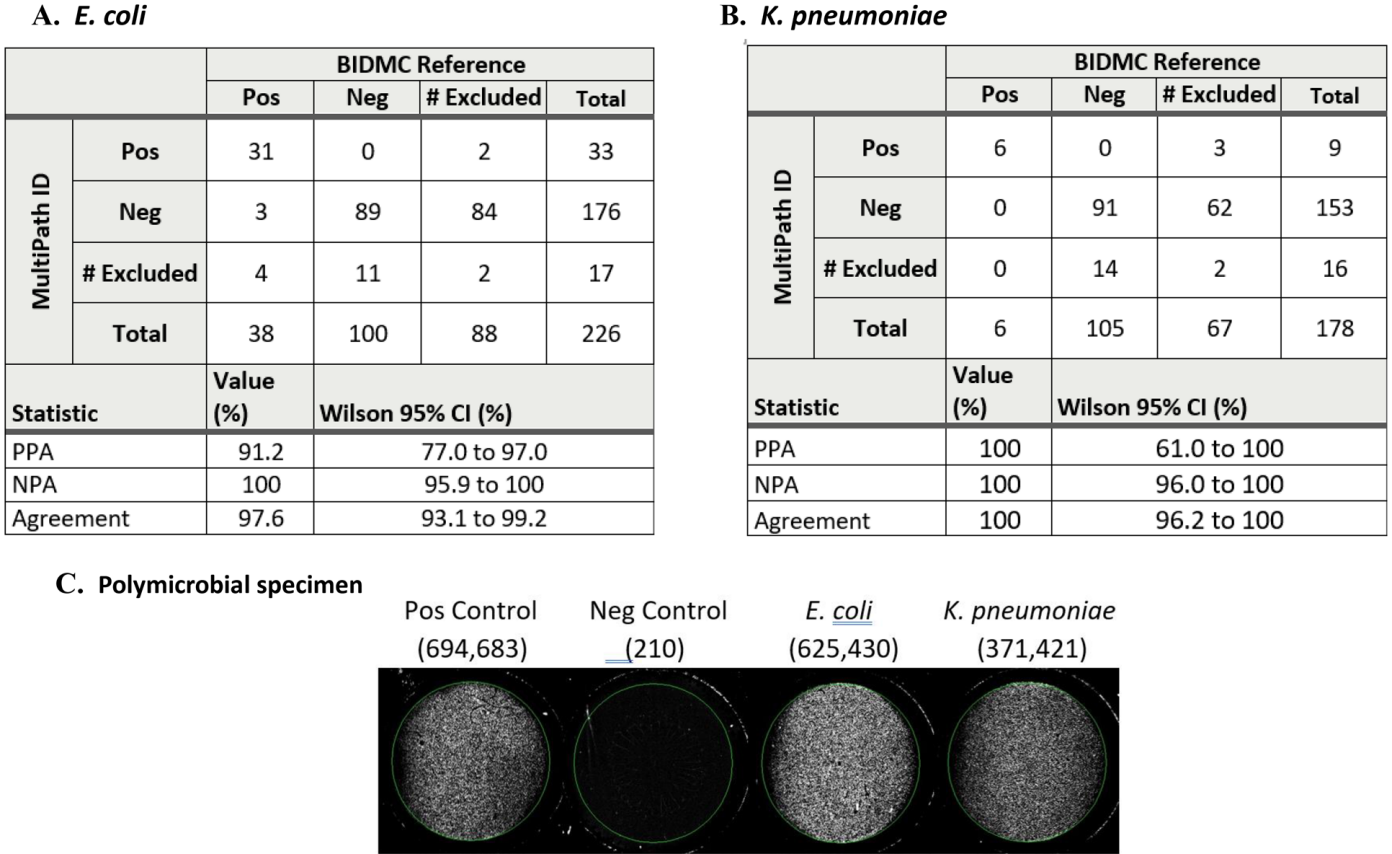
Blinded study of clinical urine specimens comparing MultiPath ID assays and BIDMC reference results (VITEK 2). (**A**) Detection of *E. coli* bacteria. (**B**) Detection of *K. pneumoniae* bacteria. Statistics show positive percent agreement (PPA), negative percent agreement (NPA) and overall agreement along with the Wilson 95% confidence intervals for proportions. (**C**) Images from the MultiPath cartridge with specimen BI703 containing both *E. coli* and *K. pneumoniae* bacteria. Left to right: positive control, negative control, *E. coli* well and *K. pneumoniae* well. The number of cells detected by MultiPath, estimated using total fluorescence, is shown in parentheses.

A blinded study was performed with deidentified, coded urine specimens (preserved in 1.8% boric acid) tested for diagnosis of suspected UTI at the Beth Israel Deaconess Medical Center (BIDMC) and provided under an IRB approved protocol (Fig. 9). 226 specimens were processed by the rapid SEC method within 48 h of collection and tested by MultiPath ID assays. 178 of these were tested by both the *E. coli* and *K. pneumoniae* MultiPath ID assays (99 in cartridges on the automated MultiPath analyzer, 79 in microwell plates) and an additional 48 specimens were tested only for *E. coli* infection (microwell plate format). After decoding, MultiPath results were compared to standard of care (SOC) clinical microbiology results (colony isolation and VITEK 2® identification) at BIDMC. The positive percent agreement (PPA), negative percent agreement (NPA) and overall agreement of MultiPath ID results relative to BIDMC SOC is presented in Fig. 9 along with 95% confidence intervals.

For *E. coli* (n = 123 valid results), the PPA, NPA, and overall agreement were 91.2%, 100% and 97.6%, respectively. The number of viable bacteria in urine specimens stored at 4 °C can decline over time, so cell concentrations at the time of MultiPath testing may have been less than at the time of SOC testing. This may explain the 3 MultiPath false negative *E. coli* results, which had colony counts close to the 10,000 CFU/mL clinical cutoff. For *K. pneumoniae* (n = 97 valid results) the PPA, NPA and overall agreement were all 100%, although the 95% confidence interval for PPA was quite wide (61.0% to 100%) because of the small n-size for positive specimens. The absence of false positive MultiPath results in clinical specimens for either ID test demonstrates the high specificity of the MultiPath FISH-based method.

The tables in Fig. 9 also show the number of results excluded from data analysis. All BIDMC reference method exclusions were due to inadequate specimens containing mixed flora (i.e., those containing 3 or more different colony morphologies) consistent with skin, genital, or fecal contamination. About 38% of the specimens from this patient population were excluded on this basis. Although BIDMC did not identify the bacterial species in these samples, the majority were negative by MultiPath ID tests and 2 of 88 (2.3%) and 3 of 67 (4.5%), were positive by MultiPath for *E. coli* and *K. pneumoniae*, respectively. The excluded MultiPath results were due to invalid internal cartridge controls (n = 4) or poor image quality in the microwell plate format. Four additional *K. pneumoniae* results were excluded based on an improper analyzer temperature setting which led to cross-reactivity with *E. coli* cells.

Specimen BI703 was identified as a polymicrobial infection by the SOC reference method containing clinically significant concentrations of both *E. coli* and *K. pneumoniae* cells. Both pathogens were also detected in BI703 by MultiPath ID tests (Fig. 9C), demonstrating that the MultiPath ID technology can accurately identify multiple pathogens in the same clinical specimen.

#### MultiPath AST accuracy in clinical specimens

542 leftover, de-identified, non-preserved urine specimens were collected from the microbiology laboratory at Lowell General Hospital (LGH) and plated on blood and MacConkey agar plates. Specimens were identified as *E. coli* positive based on morphology, lactose fermentation, hemolysis, indole production, pyrrolidonyl arylamidase (PYR) test, and cell concentrations greater than 10,000 CFU/mL. 103 (19%) were selected for AST analysis by the MultiPath *E. coli* AST test using the manual microwell plate format with 4 antibiotics at 8 concentrations each to determine MICs. Since resistance was low, an additional 30 samples were included that were contrived by adding 30 different *E. coli* strains with resistance to one or more of the 4 drugs to 30 unique negative urines obtained from LGH. The BMD method was used as the AST reference and MICs interpreted using CLSI breakpoints to establish susceptibility (Table 5).

**Table 5:** MultiPath AST accuracy vs BMD reference method for clinical specimens positive for *E. coli*.

Experimental Condition			BMD Reference			MultiPath vs BMD Reference				MultiPath	
Bacteria	Drug	n	S	I	R	CA	mE	ME	VME	MIC EA	# Invalid
*E. coli*	CIP	130	94	2	34	119	8	3	0	108	2
	CPD	129	97	0	32	125	2	1	1	118	3
	NIT	130	113	5	12	117	12	1	0	117	2
	SXT	130	84	n/a	46	118	n/a	8	4	104	2
Summary	Totals (n)	519	388	7	124	479	22	13	5	447	9
	Totals (%)	100%	74.8%	1.3%	23.9%	92.3%	4.2%	3.4%	4.0%	86.1%	1.7%

Four antibiotics were tested: ciprofloxacin (CIP), cefpodoxime (CPD), nitrofurantoin (NIT) and trimethoprim/sulfamethoxazole (SXT). AST categorical results are shown for the BMD reference method and agreement by the MultiPath method. Abbreviations and calculations of ME and VME percentages are as described in Table 4. MIC essential agreement (EA) and number of invalid MultiPath results are also shown.

One sample out of the 133 (0.8%) was excluded due to a technical issue with the preparation of a MultiPath reagent plate. Out of 528 antibiotic/sample combinations, 7 MultiPath AST results (1.3%) were invalidated due to insufficient growth without antibiotic (from 3 samples) and 2 (0.4%) were invalidated due to incongruous growth with respect to antibiotic concentration. Specimens with very high cell concentrations were diluted in PBS to about 7 × 10^5^ CFU/mL prior to SEC processing to ensure there was sufficient bacterial growth in the absence of antibiotic.

Comparison of MultiPath and BMD susceptibilities are presented in Table 5. Overall, there was 92.3% categorical agreement (CA) between the two methods for the 4 antibiotics, ranging from 90.0% for nitrofurantoin to 96.9% for cefpodoxime. Most of the 4.2% minor errors (mE) were seen with ciprofloxacin and nitrofurantoin. There were also 3.4% major errors (ME, false resistance) and 4.0% very major errors (VME, false susceptibility). The majority of ME and VME disrepancies occurred with trimethoprim-sulfamethoxazole, a 2-breakpoint drug combination known to be problematic in a variety of AST systems, including the BMD reference method, due to incomplete suppression of growth at concentrations above the MIC (so called ‘trailing growth’, where up to 20% growth is to be ignored)^21^. Similar issues were seen with this drug combination in MultiPath AST assays, where growth or growth inhibition was often inconsistent from one two-fold antibiotic dilution to the next (data not shown). Given the increased uncertainty of clearly defining the boundary between growth and no-growth by both methods for trimethoprim-sulfamethoxazole, it is not suprising that greater categorical differences were observed.

These data also show the MultiPath *E. coli* AST test is capable of generating accurate MIC values. Across all four antibiotics, 86.1% of the MultiPath MICs were within essential agreement (i.e., within +/− one twofold dilution) of the BMD MICs. The lowest agreement between MICs was seen with trimethoprim-sulfamethoxazole at 80.0%, as might be expected based on the relatively high uncertainty of precisely determining the MIC for this drug. The greatest MIC agreement was at 91.5% for cefpodoxime.

## Discussion

The first ID and AST application of MultiPath technologies is for the rapid identification of pathogens causing urinary tract infections and identification of antibiotics to which the pathogens are susceptible. These infections are especially problematic for patients at high risk for complications, including elderly patients and patients with recurrent UTIs, mainly because of the prevalence of multidrug-resistant pathogens as well as the risk of progression to urosepsis^22^. A rapid test that ensures these patients receive the optimal targeted antibiotic as early as possible in the course of their infection has the potential to improve clinical outcomes, minimize patient discomfort, improve antibiotic stewardship, and reduce healthcare costs^22^.

The studies presented were designed to test the potential of the MultiPath technology to enable rapid, accurate, and direct-from-specimen ID and AST results for patients suspected of having a UTI. The experiments address key challenges for direct-from-specimen AST including the need for high specificity, a wide dynamic range to accommodate variable cell input, robustness to urine matrix, accurate ID and AST results for targeted pathogens in the presence of commensal flora and the potential for accurate AST results for two pathogens in the same specimen. The technology combines a simple urine processing method with FISH-based specific cell labeling, magnetic cell capture and non-magnified digital imaging to count single cells and report ID results. The same technology is used to produce phenotypic AST results by comparing cell numbers after bacterial growth in the presence and absence of antibiotics. Furthermore, the MultiPath technology, including UTI ID and AST tests, has been integrated into disposable, test-specific cartridges containing all the required reagents in either dried or lyophilized form to provide room temperature test stability. Cartridges are processed on the MultiPath analyzer, a fully automated benchtop instrument with continuous random-access capability.

MultiPath 45-min ID tests have been developed for five uropathogens, including both gram-negative and gram-positive bacterial species, which account for the majority—about 88%—of uncomplicated and complicated UTI cases^15^. On the MultiPath automated system, these five ID tests showed a strong linear correlation between cells detected and input bacteria, analytical sensitivities less than 7,000 CFU/mL, and excellent precision below the clinical cutoff with CVs ranging from about 4% to 12%. Furthermore, the technology was shown to have a dynamic range of at least 4 orders of magnitude for cells in mid-log growth with no hook effect up to 1 × 10^9^ CFU/mL. The efficiency of detecting input cells was similar for the five ID tests and ranged from about 11% to 27%. These low efficiencies are due to a combination of cell loss during urine processing, cell lysis, and assay inefficiencies, suggesting that improvements in sensitivity may be possible.

MutiPath ID assays were also shown to be highly specific. Many strains of targeted bacteria were detected and all off-target species that were tested, even at high cell concentrations, were indistinguishable from no-cell controls. Furthermore, the detection of a target bacteria near the clinical cutoff was unaffected by up to a 10,000-fold excess of off-target cells. This high specificity is particularly important for direct-from-specimen MultiPath ID and AST tests where specimens may contain mixtures of uropathogens or contaminating off-target bacteria.

MultiPath AST assays produce results in under 5 h on pathogen-specific AST cartridges, including off-cartridge urine processing. Preliminary investigations showed SEC processing of urine was effective to achieve consistent pH and NaCl concentration, remove boric acid, and remove antibiotics (data not shown). Accurate MICs were achieved in spiked urine specimens from various donors, including a UTI patient taking the antibiotic under evaluation, demonstrating potential robustness to urine matrix. AST accuracy was also demonstrated in samples contrived with a 20-fold excess of commensal bacteria and for *E. coli* and *K. pneumoniae* pathogens in the same specimen, regardless of all four possible pairwise combinations of susceptible and resistant strains. Such robustness to urine matrix, AST accuracy in the presence of commensal bacteria and AST accuracy for multiple uropathogens in the same specimen are required for performing AST from specimens without first purifying single bacterial colonies. However, the impact of off-target bacteria depends on the relative growth rates and input cell concentrations of the target and off-target bacteria. These limitations have not been fully investigated. However, if the growth of target bacteria is substantially impeded by the growth of off-target cells and/or by urine matrix, this will be detected by comparing the two AST reference reactions and produce an invalid, not inaccurate, AST result.

AST accuracy was further shown for 3 pathogens, by contriving specimens in PBS and testing each with two or three antibiotics from different classes. Out of 464 results, there was 93.1% categorical agreement to the BMD reference method with only 1 ME and 2 VME. Similar results at 4 and 6 h of growth suggest, at least for these pathogen-antibiotic combinations, that accurate AST results can be achieved with only 4 h of growth.

Testing of clinical urine specimens with MultiPath ID and AST tests illustrates the promise of the rapid, direct-from-specimen MultiPath approach compared to lengthy, standard of care clinical methods. MultiPath *E. coli* and *K. pneumoniae* ID tests had 97.6% and 100% agreement to the VITEK 2 reference method, respectively, although there were just 6 positive specimens for *K. pneumoniae* which has a lower prevalence than *E.* coli in UTI patients. One specimen, containing both bacteria, was correctly identified by the MultiPath UTI ID cartridge, showing pathogen identification of polymicrobial infections is possible with this technology. MultiPath AST evaluation of 132 clinical specimens for *E. coli* with 4 antibiotics in different classes had 92.3% categorical agreement and 86.1% MIC essential agreement compared to BMD reference results. The majority of the 13 ME (3.4%) and 5 VME (4.0%) were with the troublesome drug combination trimethoprim-sulfamethoxazole. Future work will investigate this issue and will expand AST testing to include additional pathogens and antibiotics.

These studies have some limitations. UTI cartridges and internal controls were early prototypes used to demonstrate UTI tests’ performance on the MultiPath analyzer which led to exclusions of data points. In some cases, testing was limited to a small n-size or a few pathogen-antibiotic combinations; additional studies are required to confirm these initial results. Further investigations of polymicrobial infections are required since the AST polymicrobial study examined samples with two bacteria with similar input cell concentrations and growth rates. Generating a larger dataset with clinical specimens will be important to extend these initial MultiPath ID and AST results and to include pathogens other than *E. coli* and *K. pneumoniae*. Many clinical specimens from BIDMC did not have SOC species identification because they were determined to be contaminated specimens (i.e., mixed flora specimens with 3 or more species). These specimens were excluded because without a species ID, comparison was not possible to MultiPath results. However, it was noted the MultiPath technology lacked the ability to similarly identify specimens with mixed flora. This could be addressed in future MultiPath ID tests by including assays for detecting commensal flora. Mixed flora specimens would be flagged as inadequate specimens and not tested for susceptibility unless they contained one or more ‘predominant’ target pathogens (i.e., present at greater than 100,000 CFU/mL) or if requested by a clinician. Such a strategy, based on the premise that UTI pathogens are not typically seen at such high concentrations from contamination, is consistent with the current standard of care at BIDMC. Since MultiPath AST tests are largely unaffected by off-target bacteria, mixed flora specimens with a predominant pathogen are expected to yield accurate AST results for that pathogen.

Rapid antimicrobial susceptibility testing is an expanding field with many promising technologies^9–12^. Most of the rapid AST technologies, however, still require colony isolation or a pre-enrichment step (e.g., blood cultures) which typically take 1 or 2 days. Although the time to result is improved over current AST methods, at least 24 h are still required to report a result due to the necessity to use purified bacterial colonies or increase bacterial concentration by a pre-enrichment growth step. Currently, only direct-from-specimen approaches offer the promise of obtaining ID and AST results for the full range of infection and specimen types in a few hours^13,14^.

The MultiPath technology has the potential of offering clinically valuable ID and AST information within 6 h after urine specimen collection without the need for isolating colonies, particularly for patients at risk for complications if the UTI infection is allowed to progress. MultiPath ID and AST tests can generate accurate results of multiple pathogens in the same specimen and may be unaffected by off-target bacteria. Finally, the design of the MultiPath analyzer, coupled with ready-to-use consumable cartridges with internal controls and room temperature stability, makes the technology easy to use and ideal for both hospital and point-of-care settings. These advantages underlie the potential of the technologies to enable future rapid ID and AST tests for other syndromic infections including blood infections, pneumonia, surgical site infections, and wounds.

## Materials and methods

### Clinical specimens, bacterial strains/isolates, and urine

Leftover, coded, de-identified clinical urine specimens preserved in 1.8% boric acid were obtained under IRB approved protocol #2013P000059 from Beth Israel Deaconess Medical Center (BIDMC). The study was approved and the need for informed consent waived by the Committee on Clinical Investigations, the authorized Institutional Review Board and Privacy Board appointed to review research involving human subjects at BIDMC. Leftover de-identified clinical urines (unpreserved) were also obtained from Lowell General Hospital (LGH) under an agreement exempt from IRB approval at LGH by the Director of Ethics and Compliance, Compliance and Privacy Officer. Individual donor and pooled human urine—provided frozen—were purchased from Innovative Research. Urine was also donated by consenting First Light employees under IRB approved protocol #20-MDC-FLD-125 (Pearl IRB) and may have been tested fresh or after having been frozen. In all cases, HIPAA regulations were carefully followed. Bacterial strains for analytical studies were obtained from commercial biorepositories, including ATCC, CDC, ARLG and JMI Laboratories. Some *P. mirabilis*, *Enterococcus* and *K. pneumoniae* strains were kindly provided by Assurance Scientific Laboratories. Bacteria were also isolated from the leftover urine specimens from BIDMC or LGH. All work described herein was conducted in accordance with relevant guidelines and regulations.

### Contrived specimens

Samples for analytical testing were contrived by spiking either PBS or urine samples with bacterial strains at various concentrations as shown in the figures and tables. In some cases, bacteria were spiked into urine samples after SEC processing. Bacterial colonies were seeded into TSB or MHB II broth, grown to mid-log phase at 35 °C and the cell concentration in CFU/mL estimated by OD at 600 nm using pre-established conversion factors. Cultures were then diluted in either PBS or urine according to the final matrix type to the desired concentration. Starting cell concentrations were typically confirmed by plating appropriate dilutions on agar media.

### Urine processing by SEC

Clinical urine specimens, samples contrived in urine, and urine without bacteria were processed off-cartridge by a rapid SEC method. Briefly, polyacrylamide resin (Bio-Gel P-10 or Bio-Gel P-60, Bio-Rad) was hydrated for 1 min in PBS in a 5 mL column with a 35 µm lower frit, creating a bed volume where the resin was maximally absorbed but with no excess fluid. 2 mL of a urine sample was immediately added carefully onto the hydrated resin and positive air pressure (100—150 mbar) applied for 1–2 min to push the sample through the column into a collection tube.

### MultiPath assays (microwell format)

MultiPath ID tests for target bacteria were conducted in microwell plates as follows. 100 µL sample was added to a well containing either lyophilized or dried FISH reagents plus a lyophilized bead containing magnetic particles, mixed, transferred to dye-cushion plates and incubated at 35 °C for 30 min. FISH reagents were developed for each pathogen and contained an Alexa647-labeled DNA oligonucleotide probe specific to the target 16S or 23S rRNA and one or more helper oligonucleotide probes (Integrated DNA Technologies). Depending on the species, the FISH reagents also included detergent(s), chaotrope, excipient, NaCl and sodium citrate. Reagents were formulated at 4x the final concentrations and either lyophilized with excipient (e.g., 10% dextran) into 8.3 µL beads (Evik Diagnostic Innovations) or 25 µL dried in plates for 1–3 h at 60 °C. 10 µL magnetic particle beads contained about 2,500 fluorescent 0.86 µm magnetic focus particles (Bangs Laboratories), 1.4 × 10^9^ magnetic 200 nm cell-binding nanoparticles (e.g., fluidMAG-PAS, Chemicell), 50 mM EPPS buffer pH 8.2, and excipient (e.g., 10% trehalose) and were lyophilized (Evik Diagnostic Innovations). The dye-cushion plates were prepared by adding to each well 46 µL of 58 mM CAPSO pH 10, 8.1% OptiPrep density gradient medium (Sigma-Aldrich), and 5.4 mg/mL direct black 19 dye (Orient) and drying at 60 °C for 3 h. After incubation, FISH labeled cells bound to magnetic nanoparticles and fluorescent magnetic focus particles were pulled to the well’s bottom surface by placement on a magnet (Dexter Magnetic Technologies) for 4 min at 35 °C, while unbound fluorescent probe is largely excluded from the surface and rendered undetectable by the hydrated, dense dye-cushion. The Alexa647 fluorescently labeled cells were then detected by a custom, non-magnified, imager with a CMOS detector (First Light Diagnostics) using the fluorescent magnetic focus particles to obtain the correct focal plane.

MultiPath AST tests were conducted on microwell plates exactly as for ID tests described above, except 100 µL samples were first added to growth plates and incubated at 35 °C for 4 h. Growth plates were prepared by drying 25 µL of 4$\times$ MHB II or TSA media with (also at 4$\times$ concentration) or without antibiotics at 40 °C for 3 h. Following incubation, samples were transferred to a FISH reagent plate as described above for ID tests. Controls without the growth step were similarly processed to establish the input, no-growth inoculum for each condition.

### MultiPath assays (automated cartridge format)

MultiPath UTI assays were also performed automatically in 8-channel MultiPath cartridges on the MultiPath analyzer. MultiPath cartridges were configured according to each experimental design and contained barcodes to allow correct processing by the analyzer. Cartridges used to correlate MultiPath results to input cells contained a negative control in the first channel and replicate pathogen-specific assays in the other 7 channels. ID test cartridges for clinical specimens were configured with: (1) a positive control in channel 1, containing lyophilized *E. coli* cells and reagents; (2) a negative control in channel 2, formulated with probes without complementarity to bacterial rRNA; and 3) FISH reagents for specific targeted pathogens in the remaining channels.

About 1.0 mL of sample was added to the sample well of test cartridges. Cartridges were loaded into the MultiPath analyzer and automatically processed at 35 °C. The analyzer controlled all fluidic, incubation, magnetic separation, and imaging acquisition steps. Total processing time on the analyzer is about 45 min for ID cartridges and 4.75 h for AST cartridges with a 4-h growth step.

### Reference methods

Bacterial species identification or confirmation was achieved with API strips (bioMerieux), selective growth media and biochemical tests or, for clinical specimens, by standard of care microbiology at BIDMC (bioMerieux VITEK 2). Reference susceptibility results were generated by in-house BMD or disk diffusion methods. In some cases, published MICs were used but confirmed by in-house BMD or disk diffusion.

#### Imaging and data analysis

Images of fluorescently labeled cells were acquired either on the MultiPath analyzer (for cartridges) or a custom, standalone imager (for microwell plates); both used essentially identical components (First Light Diagnostics). In both cases, labeled cells were detected using LED excitation sources, optical bandwidth filter sets and a camera with a CMOS detector. Whole well images were acquired without magnification or scanning. Although the analyzer and imager have 4 color fluorescent capability, the work presented herein used one channel (Ex: 470 nm, Em: 515 nm) to detect fluorescent magnetic particles to establish the correct focal plane and another channel (Ex: 630 nm, Em: 680 nm) to detect the Alexa647-labeled bacterial cells. MultiPath software controlled the image acquisition parameters (e.g., exposure time).

Images were analyzed using MultiPath software to determine the number of fluorescently labeled objects per well and the fluorescent intensity of each object. The software includes algorithms for background subtraction and debris detection and elimination. Below 12,000 objects per imaging well, cells are directly counted and above that level, where individual objects merge, cells were estimated by dividing total fluorescence by the average fluorescent intensity per cell.

#### Results analysis

Results were analyzed using Microsoft Excel, JMP, and custom software tools. Analytical sensitivity was estimated using the upper 95% distribution of the no-cell MultiPath results and the 95% distribution of the variation of the 5000 CFU/mL input level to approximate a MultiPath cell detection limit which was then converted to CFU/mL using the ordinary least squares regression equations from the input/output correlation experiments as shown in Fig. 4.

MultiPath AST tests were considered valid if the number of cells detected in the no-abx reference was at least fivefold greater than no-growth reference. AST results were invalidated if no growth occurred at a low antibiotic concentration with growth at a higher concentration. For each bacteria/antibiotic combination, experimental data sets of strains with known MICS were used to establish growth thresholds based on relative cell counts in reactions with antibiotics compared to the no-abx reference. MICs were determined as the lowest antibiotic concentration where the relative cell counts fell below the threshold. MICs were converted to susceptibility categories (SIR) using CLSI breakpoints. In some cases, AST categorical (SIR) results were achieved without MICs by determining growth or no-growth at antibiotic concentrations at CLSI breakpoints.

## Data Availability

Raw datasets generated during and/or analyzed during the current study are not publicly available due to the proprietary nature of the image analysis algorithms but reduced datasets are available from the corresponding author upon reasonable request.
